# Vasodilation and Reduction of Systolic Blood Pressure after One Session of High-Intensity Interval Training in Patients With Heart Failure with Preserved Ejection Fraction

**DOI:** 10.5935/abc.20180202

**Published:** 2018-11

**Authors:** Juliana Beust de Lima, Anderson Donelli da Silveira, Marco Aurélio Lumertz Saffi, Márcio Garcia Menezes, Diogo Silva Piardi, Leila Denise Cardoso Ramos Ramm, Maurice Zanini, Rosane Maria Nery, Ricardo Stein

**Affiliations:** Hospital de Clínicas de Porto Alegre - Universidade Federal do Rio Grande do Sul, Porto Alegre, RS - Brazil

**Keywords:** Heart Failure, Arterial Pressure, Exercise, Vasodilatation, Brachial Artery, Endothelium/function

## Abstract

**Background:**

Heart failure with preserved ejection fraction (HFpEF) is a multifactorial syndrome characterized by a limited exercising capacity. High-intensity interval training (HIIT) is an emerging strategy for exercise rehabilitation in different settings. In patients with HFpEF, HIIT subacute effects on endothelial function and blood pressure are still unknown.

**Objective:**

To evaluate the subacute effect of one HIIT session on endothelial function and blood pressure in patients with HFpEF.

**Methods:**

Sixteen patients with HFpEF underwent a 36-minute session of HIIT on a treadmill, alternating four minutes of high-intensity intervals with three minutes of active recovery. Brachial artery diameter, flow-mediated dilation, and blood pressure were assessed immediately before and 30 minutes after the HIIT session. In all analyses, p <0.05 was considered statistically significant.

**Results:**

There was an increase in brachial artery diameter (pre-exercise: 3.96 ± 0.57 mm; post-exercise: 4.33 ± 0.69 mm; p < 0.01) and a decrease in systolic blood pressure (pre-exercise: 138 ± 21 mmHg; post-exercise: 125 ± 20 mmHg; p < 0.01). Flow-mediated dilation (pre-exercise: 5.91 ± 5.20%; post-exercise: 3.55 ± 6.59%; p = 0.162) and diastolic blood pressure (pre-exercise: 81 ± 11 mmHg; post-exercise: 77 ± 8 mmHg; p = 1.000) did not change significantly. There were no adverse events throughout the experiment.

**Conclusions:**

One single HIIT session promoted an increase in brachial artery diameter and reduction in systolic blood pressure, but it did not change flow-mediated dilation and diastolic blood pressure.

## Introduction

Heart failure with preserved ejection fraction (HFpEF) is a complex and prevalent clinical syndrome characterized by a significant limitation to exercising capacity, and pharmacological treatment has not evidenced any improvement in mortality rates in this scenario yet.^1^^,^^2^ Therapeutic approaches are limited and they are mainly based on symptom management and control of cardiovascular risk factors, such as high blood pressure (BP).^3^^-^^5^

Hypertension is associated with increased oxidative stress and vascular inflammation, closely related to endothelial dysfunction.^6^^,^^7^ On the other hand, attenuated endothelial function in individuals with HFpEF contributes to intolerance to exercising^8^^-^^10^ and it is an independent predictor of adverse cardiovascular events.^11^^,^^12^ As a non-pharmacological intervention, exercise training appears as a potential strategy to be included in HFpEF's therapeutic arsenal.^13^^,^^14^

High-intensity interval training (HIIT) has emerged as an exercise modality with a positive impact on some cardiovascular outcomes, and it is at least as effective as moderate-intensity continuous training in patients with heart failure with reduced ejection fraction.^15^^-^^17^ Recent meta-analyses have demonstrated that HIIT, in a long-term basis, is more effective in promoting endothelial function improvement and BP reduction in individuals with cardiovascular risk factors.^18^^,^^19^ In previous studies, after one single HIIT session, patients with coronary artery disease and hypertension showed increased brachial artery diameter,^20^^,^^21^ improved endothelial function,^20^ and reduced BP.^21^^-^^23^

It is well known that HFpEF patients have attenuated vasodilator reserve while exercising and their ventricular-arterial coupling responses are impaired.^9^^,^^10^^,^^24^ However, the effect of one HIIT session on endothelial function and BP in these patients is still unknown. Considering this gap in the literature, the aim of this study was to evaluate brachial artery diameter, endothelial function, and BP 30 minutes after one HIIT session in patients with HFpEF.

## Methods

### Study design and patients

This before-and-after (quasi-experimental) study was conducted between June 2014 and November 2015. Nineteen patients with HFpEF, according to the European Society of Cardiology criteria,^25^ were sequentially recruited in an outpatient cardiology clinic of a tertiary hospital in southern Brazil. Eligibility criteria were presence of signs and symptoms of heart failure, preserved ejection fraction (> 50%), diastolic dysfunction (left ventricular end-diastolic volume index < 97 mL/m^2^) with increased filling pressure (E/e' > 8), and in the case of E/e' < 15, at least one diagnostic criterion for HFpEF, according to the abovementioned document. Age between 40-75 years, New York Heart Association (NYHA) functional class I to III, and clinical stability under optimal drug therapy in previous 3 months, was also considered criteria for eligibility. Patients with severe lung disease, moderate-to-severe valvular disease and peripheral arterial disease were excluded. Similarly, autonomic neuropathy, unstable angina, a history of complex arrhythmias induced by stress, patients with implantable cardiac electronic devices and those with cognitive and/or limiting musculoskeletal conditions, were excluded.

Firstly, patients underwent a Doppler echocardiography with color flow mapping to confirm the diagnosis criteria for HFpEF. Then, a maximal cardiopulmonary exercise testing was performed to assess ventilatory thresholds and peak oxygen consumption, as well as heart rate response to exercise. Up to 14 days after the cardiopulmonary exercise testing, brachial artery diameter, flow-mediated dilation (FMD) and endothelium-independent dilation were assessed immediately before and 30 minutes after a HIIT session. In the same experimental session, BP and heart rate were measured at two different moments before and after exercise as described below.

### Measurements and instruments

#### Patients' characteristics at baseline

Demographic and clinical data were collected on the first day through a questionnaire and verified in the medical records of each patient. Anthropometric data were collected at the time the questionnaire was completed.

#### Transthoracic echocardiogram

All echocardiographic examinations were performed using equipment Envisor C HD or HD 11 (Philips, USA) with a standard multifrequency sectorial transducer by a trained cardiologist. Images were acquired following a standardized protocol, following recommendations present in the current guidelines.^25^^,^^26^ Cine loops and static images of 3 consecutive beats were recorded on standard 2D, M-mode, Doppler and tissue Doppler echocardiographic views. Left ventricular ejection fraction was calculated using the Teichholz formula from the parasternal long-axis view. For patients with regional wall motion abnormalities, the Simpson rule was used to calculate the ejection fraction. Left atrium volume was measured at ventricular systole, just before mitral valve opening, and calculated from apical 4-and 2-chamber views using the biplane method of disks. Left ventricular diastolic function was evaluated with transmitral pulsed Doppler (peak E velocity, peak A velocity, E/A ratio and deceleration time) and mitral annulus tissue Doppler velocity (early diastolic velocity - e', late diastolic velocity - a').

#### Cardiopulmonary exercise test

The test was performed on a treadmill (General Electric T-2100, GE Healthcare, Waukesha, USA), and breath-by-breath expired gas analysis was carried out using a Cortex Metalyzer 3B system (Cortex Medical, Leipzig, Germany). Heart rate was monitored with a 12-lead electrocardiograph (Nihon Kohden Corporation, Tokyo, Japan), with electrode placement as described by Mason and Likar.^27^ BP was measured with a sphygmomanometer (PA 2001, P.A. MED, São Paulo, Brazil) every 3 minutes during the test and also at the physician's discretion. All tests were performed in the morning, with room temperature between 18 and 22ºC and relative humidity around 60%, and they were conducted always by the same researcher (ADS), a cardiologist with expertise in cardiopulmonary exercise testing, certified by the Department of Exercise Testing and Cardiovascular Rehabilitation of the Brazilian Society of Cardiology. An individualized ramp protocol was used as described elsewhere in this study.^28^ Tests were considered maximal when the respiratory quotient (R) was equal to or higher than 1.10.

### Blood pressure

BP was measured with a digital device (G-Tech MA100, Shenzen, China) at four different points in time: 1) pre-assessment of endothelial function (after 15 minutes seating at rest); 2) immediately before HIIT session; 3) 5 minutes after HIIT session; 4) 30 minutes after HIIT session.

### Endothelial function

Patients were instructed not to do any type of exercise, not to smoke, and not to dear or drink any caffeine or alcohol for 24 hours before the evaluation. The evaluation started after 15 minutes of seated rest in a room with temperature between 18 and 22ºC. Patients stood in the supine position with their left arm positioned comfortably. Noninvasive measurements of endothelial function were performed using a two-dimensional Philips EnVisor Ultrasound system (Philips, USA) with an electrocardiogram module and a high-frequency (7-12 MHz) vascular transducer.

An image of the brachial artery was obtained 2-5 cm from the antecubital fossa on a longitudinal plane. Artery diameter was manually measured from the anterior and posterior intimal layer. Visual inspection of single frames was performed and calipers were placed at discrete points along the long axis of the B-mode image, when means were calculated.

After measurements of brachial artery diameter were taken at baseline, a sphygmomanometer was inflated on patient's left forearm with 50 mmHg above the systolic BP, remaining there for 5 minutes. Sixty seconds after deflation of the sphygmomanometer cuff, a new image was recorded synchronized with the R wave of the electrocardiogram to identify the artery diameter, enabling FMD measurements.

After 15 minutes (for normalization), the artery diameter was measured again. Then, a dose (0.4 mg) of nitroglycerin spray was administered sublingually. After 5 minutes, another image was recorded to measure endothelium-independent dilation. These data were obtained before exercise and 30 minutes after the HIIT session.

FMD was expressed as the relative change in brachial artery diameter during the hyperemic phase, as follows: [(post-hyperemic diameter - baseline diameter) / baseline diameter] × 100.

### High-intensity interval training protocol

The HIIT session was performed on a treadmill according to the protocol recommended by the European Society of Cardiology (ESC).^15^ The session started with an 8-minute warm-up at moderate intensity followed by four blocks of 4 minutes each at 85-95% maximal heart rate, 15 to 17 on Borg rating of perceived exertion scale,^29^ alternated with 3 minutes at 60-70% maximal heart rate, 11 to 13 on Borg scale. It ended with 3 minutes of cool-down at moderate intensity, totaling 36 minutes. The heart rate target zone stipulated for each block was based on the maximal heart rate reached at cardiopulmonary exercise testing and was continuously measured during training through 12-lead electrocardiographic monitoring (Nihon Kohden Corporation, Tokyo, Japan).

### Statistical analysis

Data were analyzed using SPSS, version 20.0. Categorical variables are described as absolute frequencies and percentages. Continuous variables with normal distribution are described as means and standard deviations. The only variable without normal distribution (VE/VCO_2_ slope) was described as median and interquartile range. After meeting the assumptions of normality, the Student t-test for paired samples was used to compare means of the endothelial function variables (brachial artery diameter, FMD, and endothelium-independent dilation) pre- and post-exercise. Generalized estimating equations (GEE) were used to compare mean BP and heart rate between four different moments during the experiment. In all analyses, p <0.05 was considered statistically significant.

## Results

**Figure 1:**
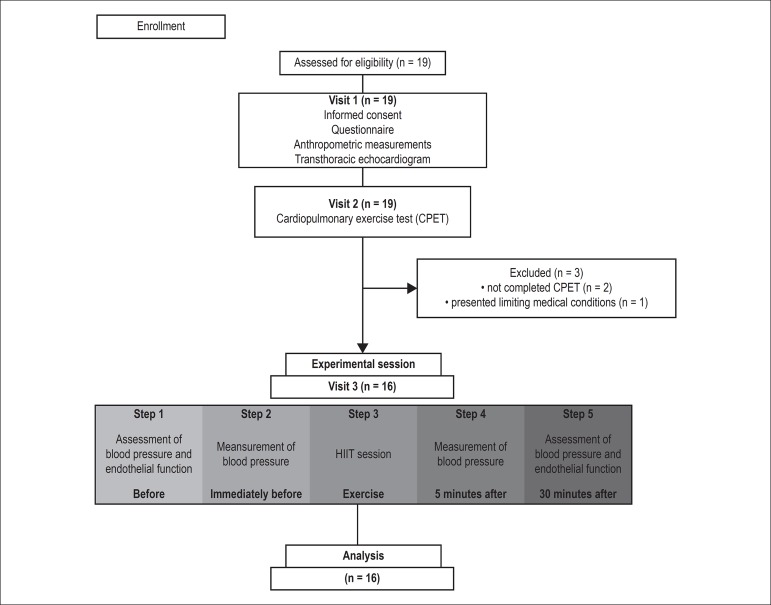
Study flow diagram.

Initially nineteen patients were included in the study. After the first evaluation, two patients who did not complete the cardiopulmonary exercise testing and one who had a limiting medical condition were excluded, as shown in Figure 1.

Table 1 shows the demographic, anthropometric, and clinical characteristics of the sample.

**Table 1:** Participants’ characteristics at baseline

Characteristic	n = 16
Female	9 (56%)
Age (years)	59 ± 7
Weight (kg)	87 ± 28
Height (cm)	159 ± 10
Body mass index (kg/m^2^)	34 ± 7
Waist circumference (cm)	110 ± 27
**Smoking**	
Active smoker	2 (12%)
Former smoker	7 (44%)
**NYHA functional classification**	
II	12 (75%)
III	4 (25%)
**Comorbidities**	
Hypertension	16 (100%)
Diabetes	7(44%)
Rheumatic disease (gout)	2 (12%)
Atrial fibrillation	1 (6%)
CRF	4 (25%)
AMI	2 (12%)
Stroke	3 (19%)
**Medications**	
ACEI/ARA	16 (100%)
Beta-blockers	13 (81%)
Diuretics	13 (81%)
Calcium channel blockers	11 (69%)
Statins	10 (62%)
Antiplatelets	9 (56%)
Vasodilators	7 (44%)
Hypoglycemic drugs	7 (44%)

Values are described as mean ± standard deviation or absolute frequency (percentage). Former smoker: more than 1 year without smoking; NYHA: New York Heart Association; CRF: chronic renal failure; AMI: acute myocardial infarction; ACEI: angiotensin-converting enzyme inhibitors; ARA: angiotensin receptor antagonists.

All patients presented normal ejection fraction, reduced left ventricular end-diastolic volume index and increased filling pressure, as shown in table 2. However, eight patients presented 15 > E/e' > 8. Among these individuals at least one diagnostic criterion for HFpEF was confirmed. Reduced functional capacity and increased ventilatory inefficiency were identified by cardiopulmonary exercise testing. The mean peak respiratory exchange ratio > 1.1 was reach as maximality criterion as shown in table 3.

**Table 2:** Echocardiographic variables

Variables	n = 16
LVEF (%)	68 ± 5
E/e’	13 ± 4
LAD (cm)	4.22 ± 0.41
LVESV (ml)	37.9 ± 9.10
LVEDV (ml)	124.41 ± 23.24
LVEDVI (ml/m^2^)	67.09 ± 6.35
IVST (cm)	1.15 ± 0.17
PWT (cm)	1.10 ± 0.19
LVM (g)	244.35 ± 58
LVMI (g/m^2^)	146.2 ± 35.84
LAVI (ml/m^2^)	20.81 ± 3.40

Values are described as mean ± standard deviation.LVEF: left ventricular ejection fraction; E/e’: early diastolic peak velocity and diastolic peak velocities of the mitral annulus ratio; LAD: left atrium diameter; LVESV: left ventricular end-systolic volume; LVEDV: left ventricular end-diastolic volume; LVEDVI: left ventricular end-diastolic volume indexed to body surface; IVST: interventricular septum thickness; PWT: posterior wall thickness; LVM: left ventricular mass; LVMI: left ventricular mass indexed by body surface; LAVI: left atrial volume indexed to body surface.

**Table 3:** Cardiopulmonary exercise testing variables

Variables	n = 16
VO_2_ peak (mL.kg^-1^min^-1^)	18.40 ± 3.16
HR max. (bpm)	125 ± 23
VE/VCO_2_ slope	33 ± 6
PET CO_2_ rest (mmHg)	33 ± 3
Pulse O_2_	11.36 ± 4.45
R peak	1.16 ± 0.13

Values are described as mean ± standard deviation or median±interquartile range. VO_2_ peak: peak oxygen consumption; HR max.: maximum heart rate; VE/VCO_2_ slope: incline of the ventilatory equivalent of carbon dioxide; PET CO_2_ rest: expired pressure of carbon dioxide; O_2_ pulse: oxygen pulse; R peak: respiratory quotient.

All patients tolerated exercise and completed the experimental session. Exercise protocol variables are described in table 4.

**Table 4:** Exercise protocol variables

Variables	Moderate intensity	High Intensity
HR (bpm)	98 ± 19	113 ± 24
BORG	13 ± 2	16 ± 2
Speed (km/h)	3 ± 0.3	4.9 ± 0.8
Incline (%)	0.9 ± 0.9	5.5 ± 1.9

Values are described as mean ± standard deviation. HR: heart rate; BORG: scale of perceived exertion.

**Figure 2:**
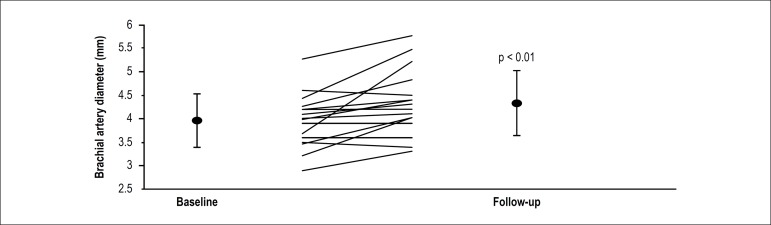
Brachial artery diameter pre- and post-high-intensity interval training session. Data are expressed as mean± standard deviation. Lines represent individual values. Probability value indicates within-group significant differences.

One single HIIT session promoted subacute increase of 0.37 ± 0.44 mm in brachial artery diameter, as shown in Figure 2. This increase was also observed in brachial artery diameter post-hyperemia. However, when these data were used to calculate pre- and post-HIIT variation in the artery diameter, there was no difference in absolute FMD and relative FMD. Also, there was no difference in the brachial artery diameter pre-NTG (Nitrogen) and post-NTG. Similarly, there was no difference in absolute endothelium-independent dilation and relative endothelium-independent dilation after one HIIT session, as presented in table 5.

**Table 5:** Brachial artery diameters and variations pre- and post-high-intensity interval training session.

Variables	Pre	Post	p
Brachial artery diameter (mm)	3.96 ± 0.57	4.33 ± 0.69	< 0.01
Brachial artery diameter post-hyperemia (mm)	4.19 ± 0.61	4.47 ± 0.66	< 0.05
Absolute FMD (mm)	0.23 ± 0.20	0.13 ± 0.26	0.177
Relative FMD (%)	5.91± 5.20	3.55 ± 6.59	0.162
Brachial artery diameter pre-NTG (mm)	4.11 ± 0.65	4.16 ± 0.68	0.528
Brachial artery diameter post-NTG (mm)	4.57 ± 0.65	4.52 ± 0.64	0.541
Absolute NTG (mm)	0.46 ± 0.17	0.35 ± 0.20	0.106
Relative NTG (%)	11.4 ± 4.4	9.0 ± 5.37	0.117

Values are described as mean ± standard deviation. FMD: flow-mediated dilatation; NTG: nitroglycerin.

**Figure 3:**
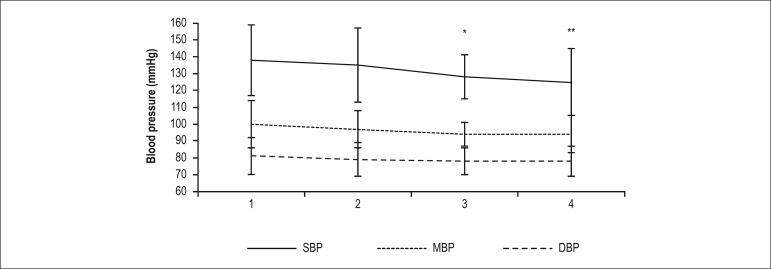
Variation of blood pressure pre- and post-high-intensity interval training session. Data are expressed as mean± standard deviation. Lines represent mean values:.1) pre-assessment of endothelial function; 2) immediately before HIIT session; 3) 5 minutes after HIIT session; 4) 30 minutes after HIIT session. SBP, systolic blood pressure; MBP, mean blood pressure; DBP, diastolic blood pressure. Probability value indicates within-group differences between points 3 and 1, and points 4 and 1 of SBP. *p < 0.05, **p < 0.01.

Baseline systolic and diastolic BP were 138 ± 21 mmHg and 81 ± 11 mmHg, respectively. Figure 3 shows variation in BP at four different points in time of the experiment. A significant reduction in systolic BP was observed 5 and 30 minutes after the HIIT session compared to the first measurement. There was no difference in diastolic BP and mean BP before and after the HIIT session.

## Discussion

To our knowledge, this is the first study to show that one single session of HIIT is effective in promoting a significant subacute increase in brachial artery diameter, which was accompanied by a significant reduction in systolic BP in patients with HFpEF. Borlaug et al.^9^ demonstrated that these individuals have global dysfunction in cardiovascular reserve, showing an impaired reduction in systemic vascular resistance and a blunted increase in blood flow while exercising. According to the authors, these phenomena are potential contributors to limited functional capacity in this situation.

Patients with HFpEF in our sample showed vasodilation after one single HIIT session suggesting that this type of exercise is a stimulus capable of promoting subacute systemic vasomotor changes, even in patients with impaired ventricular-arterial coupling^9^^-^^11^ and chronic vascular dysfunction.^6^^,^^8^^,^^30^ It is important to mention that some acute and subacute physiological responses to exercise may be clinically relevant. These responses can be superimposed after consecutive exercise sessions are carried out as a temporal summation and they may contribute to chronic adaptations of exercise training.^31^ Thus, successive sessions of exercise that increase blood flow, shear stress and, consequently, bioavailability of nitric oxide, may be a key mechanism for chronic adaptations in peripheral hemodynamics.^32^ Fu et al. found that after 12 weeks of HIIT, patients with HFpEF increased the VO_2_peak and improved peripheral hemodynamics, through increased blood distribution and oxygen extraction by the musculature while exercising.^33^

Exercise-mediated increases in shear stress have a strong and dose-dependent effect on conduit artery dilation.^33^ Birk et al.^34^ observed that vasodilation occurred in a greater extent immediately after highly intensive exercising compared to lowly intensive exercise sessions.^34^ However, it seems that the greater the vasodilation promoted by exercise, the lower the vasodilating response observed by occlusion immediately after the exercise session in healthy individuals.

Although there is no previous publication concerning subacute effect of an exercise session on endothelial function in patients with HFpEF, previous studies have evaluated patients with heart failure with reduced ejection fraction in a similar context.^35^^,^^36^ Those participants responded to a single cycling exercise session with improved forearm endothelium-dependent vasodilation (reactive hyperemia) evaluated by plethysmography up to 30 minutes after exercise.^35^ Currie et al.^20^ evaluated coronary artery disease patients after one single HIIT session and found an increase in the endothelial function after 60 minutes.^20^ In other experiment, the same group showed that only individuals with coronary artery disease with endothelial dysfunction presented augmentation in FMD after 15 minutes of a HIIT session.^21^ Interestingly, as in our experiment, in both studies the brachial artery diameter was increased.

Some evidence points out that exercising performed at submaximal intensities closer to the peak of exercise promotes a greater and longer reduction in BP after exercising than when exercising less intensively.^37^^,^^38^ The hypotensive effect of HIIT is already well established in the literature, but prior to this study, BP had not been evaluated in patients with HFpEF after a session of any type of exercise. In our experiment, we observed an absolute reduction of 12.7 ± 3.8 mmHg in systolic BP 30 minutes after an exercise session. On a chronic basis, this reduction may have clinical relevance, especially in the case of a syndrome whose strict control of BP pressure is crucial. Interestingly, a recent meta-analysis has demonstrated that HIIT performed at least 3 times a week for 12 weeks resulted in a significant reduction in systolic BP in overweigh/obese individuals.^19^

It is noteworthy that in this subgroup of individuals with HFpEF and reduced functional capacity, high-intensity exercising was well tolerated, once appropriate overload (speed and slope) was individually prescribed, always considering the target zones established based on maximal cardiopulmonary exercise test results of each individual.

Finally, in a condition characterized by exercise limitation, aerobic exercise training has a significant role and is indicated for all patients capable of performing it. In an acute and subacute setting, HIIT reduced BP and increased brachial artery diameter, suggesting that this training modality could be a beneficial alternative for individuals with HFpEF.

### Limitations and future perspectives

This was a small, single-center; before-and-after study with HEpEF patients where the presence of diabetes, atherosclerosis, gout, and use of tobacco may have influenced the study outcomes. However, these characteristics represent the reality of this complex syndrome which have multiple comorbidities. We acknowledge that further studies are necessary to evaluate the effect of a HIIT session, especially after one hour, as well as the long-term efficacy of this exercise strategy as part of a cardiovascular rehabilitation program for these patients. Finally, the presence of a control group of matched individuals without HFpEF could help establishing which responses can be attributed to the syndrome under study. Likewise, comparing a HIIT session with a continuous moderate-intensity training session could help establishing the differences in hemodynamic response among these different exercise protocols.

## Conclusion

One single HIIT session promoted an increase in brachial artery diameter and a reduction in systolic BP, and did not change FMD and diastolic BP 30 minutes after the exercise session.
